# A comparison between acupotomy vs the local steroid injection for the management of soft tissue disorder: A systematic review protocol

**DOI:** 10.1097/MD.0000000000017926

**Published:** 2019-11-11

**Authors:** Yifeng Shen, Tao Cai, Ting Li, Juan Zhong, Jing Guo, Huarui Shen

**Affiliations:** a Hospital of Chengdu University of Traditional Chinese Medicine; b Chengdu Sport University, Chengdu; c College of Pharmacy, Southwestern Medical University; d Department of Joint Surgery, Affiliated Traditional Chinese Medicine Hospital of Southwest Medical University, Luzhou City, Sichuan Province, PR China.

**Keywords:** Acupotomy, protocol, soft tissue disorder, systematic review

## Abstract

**Background:**

This systematic review protocol aims to provide the methods used to evaluate the effectiveness of acupotomy therapy for treating soft tissue disorder comparing to local steroid injection.

**Methods:**

Fifteen databases will be searched from inception to Dec 2019. We will include randomized controlled trials (RCTs) assessing acupotomy for soft tissue disorder. All RCTs on acupotomy or related interventions will be included. Study inclusion, data extraction and quality assessment will be performed independently by two reviewers. Assessment of risk of bias and data synthesis will be performed using RevMan 5.3 software. Cochrane criteria for risk-of-bias will be used to assess the methodological quality of the trials.

**Results:**

This study will provide a high-quality synthesis of pain visual analog scale and functional disability or the quality of life, the success treatment rate, the recurrent rate, and the complications rate to assess the effectiveness and safety of acupotomy for soft tissue disorder patients compare to local steroid injection.

**Conclusion:**

This systematic review will provide evidence to judge whether acupotomy is an effective intervention for patients with soft tissue disorder.

**PROSPERO registration number:**

CRD42018109080.

## Abstract 2

Supplemental Digital Content is available in the text

## 1 Introduction

Soft tissue disorders are medical conditions affecting soft tissue. Often soft tissue injuries are some of the most chronically painful and difficult to treat because it is very difficult to see what is going on under the skin with the soft connective tissues, fascia, joints, muscles and tendons. Soft tissue musculoskeletal pain syndromes manifest in isolation or secondary to underlying mechanical derangements or systemic inflammatory disease. Musculoskeletal complaints account for up to 30% of all primary care office visits. Common causes of soft tissue musculoskeletal pain include tendinitis, enthesitis, and bursitis.^[1]^

These disorders can be diagnosed by history and physical examination. Therapy typically requires a combination of avoiding the aggravating activity, education, and physical therapy. Analgesics and NSAIDs should be used judiciously. Corticosteroid injections and surgical options should be considered only when conservative therapy is ineffective.^[2]^ Corticosteroids have played a standard role in the multimodal pain management in the treatment of chronic spinal pain (cervical and lumbar) and osteoarthritis pain over the past 3 decades.^[3]^

Acupotomy, also referred to as mini-scalpel needle or needle-knife, is 1 complementary and integrative medicine modality that modernizes acupuncture by combining conventional acupuncture needle and small-knife.^[4]^ It has been used as a tool for minimally invasive operative management for decades. The origin of the treatment is “Nine Classical Needles” from the era of Huangdi's Internal Classic (Huangdi's Internal Classic, Huang Di Nei Jing); the treatment was developed into a modernized tool, acupotomy, by Zhu Hanzhang in 1976.^[5]^ Nowadays, Acupotomy has been widely used clinically by doctors of traditional Chinese Medicine, orthopedics and pain department to treat soft tissue disorder China with satisfied efficacy.^[6–9]^ Korean scholars also introduced acupotomology into clinical treatment.^[10,11]^ Acupotomy is widely used for musculoskeletal conditions, clinical evidence suggests that this treatment can relax muscular spasm and relieve compressed nerves and vessels by using the small-knife to detach taut muscle bands.^[12,13]^

However, the effectiveness of acupotomy for soft tissue disorder remains controversial. Although a meta-analysis study of the effects of various types of acupuncture in myofascial pain syndrome, acupotomy was a superior modality for improving the pressure pain threshold when compared to manual acupuncture, electro-acupuncture, dry-needling, acupuncture point injection, and fire-needle.^[14]^ The comparison and systematic review of the acupotomy is mostly compared with the traditional Chinese medicine method,^[15–18]^ others may not recognize it as such.

This study adopts the method of evidence-based medicine to analyze and evaluate clinical RCTs in patients with soft tissue disorder. In this study, we used internationally recognized local steroid injection therapy as a control and included more disease types and international literatures, in order to eliminate the impact of low-quality literature and obtain more evidence-based results.

## 2 Methods

### 2.1 Inclusion criteria for study selection

#### 2.1.1 Types of studies

All the RCTs of acupotomy for the management of soft tissue disorder patients will be included without publication status restriction or writing language.

#### 2.1.2 Types of patients

Inclusion criteria for study populations will be all patients with soft tissue disorder. No restrictions will be applied in terms of age, sex or ethnicity.

#### 2.1.3 Types of interventions and controls

Interventions to be examined will include treatment with acupotomy (there is no limit on the needle materials, treatment methods, and course of treatment) as the sole intervention, and comparator will only adopt the local steroid injection. No language restrictions will be imposed.

#### 2.1.4 Types of outcome measures

*Primary outcomes*: Improvement in pain, as measured by the visual analogue scale (VAS) or other validated pain scoring system if VAS is not used.

*Secondary outcomes*: The secondary outcomes are reduction in other scales or questionnaires evaluating pain or functional disability or the quality of life; The success treatment rate (after treatment the participants with a reduction of scales =50% comparing to baseline), the recurrent rate and the complications rate.

### 2.2 Search methods for the identification of studies

#### 2.2.1 Data sources

Electronic databases will be searched from their inception and will include Cochrane Central Register of Controlled Trials, PubMed, MEDLINE, EMBASE, and 4 Chinese databases (China National Knowledge Infrastructure, Chinese Biomedical Literature Database, VIP Database and Wanfang Database), 6 Korean databases (Korean Studies Information, DBPIA, Korean Institute of Science and Technology Information, KERIS, KoreaMed, Korean National Assembly Library) and the Japanese database (CiNii Articles). We will also conduct non-electronic searches of conference proceedings, our own article files. The search strategy that will be applied in the MEDLINE database is presented in Appendix A. Similar search strategies will be used in the other databases. We will also search the reference lists of review articles and identify RCTs for any possible titles matching the inclusion criteria.

#### 2.2.2 Searching other resources

The authors will scan the reference lists and retrieve additional studies. In addition, authors will search the WHO International Clinical Trials Registry Platform (ICTRP) (http://apps.who.int/trialsearch/) and Google Scholar (http://scholar.google.co.kr/). Dissertations of degrees will be included. The ClinicalTrials.govregistry (http://clinicaltrials.gov/) will be searched for any unpublished trials.

### 2.3 Data extraction, quality and validation

#### 2.3.1 Study inclusion

**Figure 1:**
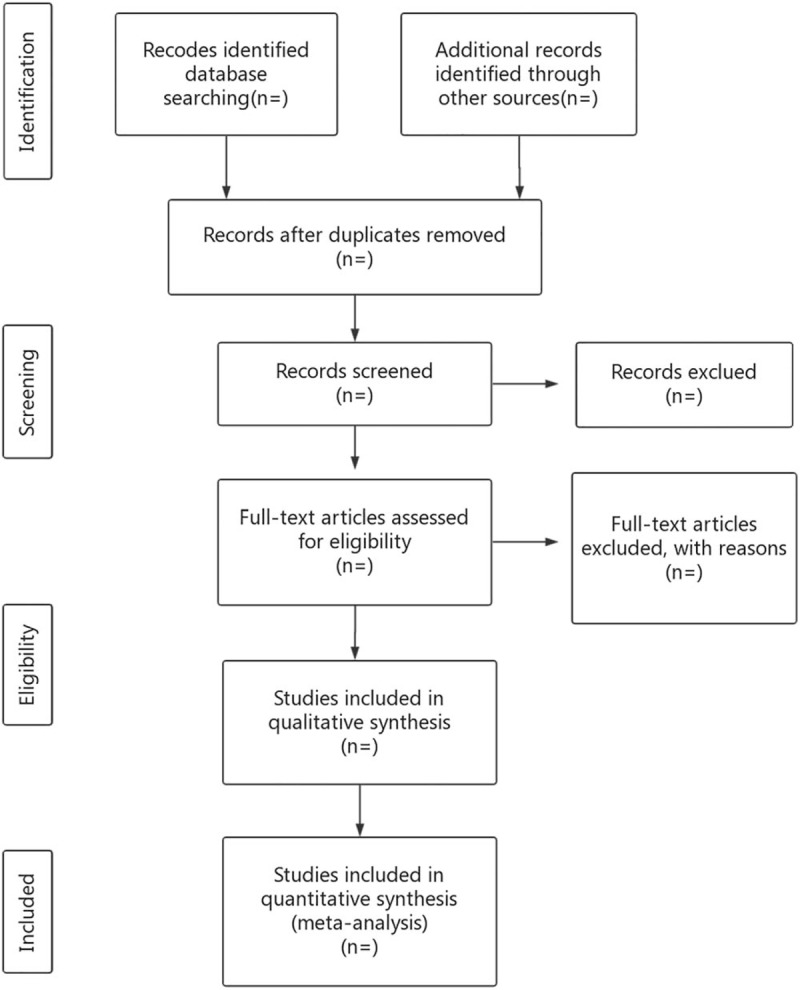
Flow diagram of study selection process.

Researchers will import the literature retrieved to the EndnoteX7 and eliminate the duplicate data. The noticeably below-standard articles will be deleted by reading the title and abstract. After that, the researchers will read the full text, discuss in the group, and contact the author for research details to determine the final inclusion of the literature (Fig. 1). The final list of articles will be converted into Microsoft Excel format. Two researchers will independently conduct the literature search and literature screening. Finally, another study member will resolve the inconsistencies and check the final literature that will be included.

#### 2.3.2 Data extraction and management

Data from the selected articles will be extracted and filled by 2 reviewers independently in the data extraction form. Any disagreement will be solved by consensus or an arbiter. We will extract information such as reference ID, author, time of publication, characteristics of participants, blinding, interventions, follow-up, outcome indicators, research results, adverse events, and other detail information. We will be in contact with the authors of trials for further information when necessary.

### 2.4 Risk of bias assessment

The risk of bias will be evaluated by 2 reviewers based on the Cochrane collaboration's tool from 7 dimensions: random sequence generation, allocation concealment, the blinding method for patients, researchers and outcomes assessors, incomplete result data, and selective reports. The terms ”Low“, ”Unclear“, and ”High“ will be referred to low, uncertain, and high risks of bias, respectively. In most cases, disagreements will be settled by discussion between the 2 reviewers. If disagreement remained after discussion, a third reviewer will be consulted before taking the final decision on the disagreements.

### 2.5 Quantitative data synthesis and statistical methods

#### 2.5.1 Quantitative data synthesis

In our review, meta-analysis will be performed using software RevMan 5.3. For dichotomous data, we will present results as risk ratio (RR) with 95% confidence intervals (CIs). For continuous data, mean difference (MD) will be included in the meta-analysis. If outcome variables are measured on different scales, standard mean differences (SMD) analysis with 95% CIs will be included in the meta-analysis.

#### 2.5.2 Assessment of heterogeneity

The heterogeneity of the research results will be analyzed through *x*^2^ test (*a* = 0.1) and determined by an *I*^2^ value. If *I*^2^ < 50%, the statistic heterogeneity among trials can be negligible, and the effect size will be estimated using the fixed-effects model. If *I*^2^ > 50%, then there is a significant heterogeneity among the trials.

#### 2.5.3 Assessment of reporting biases

If a sufficient number of studies are available (at least 10 studies), we will attempt to assess publication bias using a funnel plot.

#### 2.5.4 Subgroup analysis and investigation of heterogeneity

If there is a significant heterogeneity in the included trials, we will conduct subgroup analysis based on the type of disease, acupotomy and corticosteroid injection, differences in treatment frequencies and follow-up durations will also be included.

#### 2.5.5 Sensitivity analysis

If the test for heterogeneity *P* value is less than 0.1 after performing the subgroup analysis, the sensitivity analysis will be conducted to evaluate the robustness of our results. The meta-analysis will be repeated after omitting the low-quality studies. Moreover, we will also assess whether the statistical model (random-effects vs fixed-effects model) will affect the current results.

#### 2.5.6 Grading the quality of evidence

We will apply the Grading of Recommendations Assessment, Development and Evaluation (GRADE) method to evaluate the level of confidence in regards to outcomes. Two independent reviewers will conduct the assessment. In most cases, disagreements were resolved by discussion between the two reviewers. If disagreement remained after discussion, a third reviewer will be consulted before taking the final decision on the disagreements.

## 3 Discussion

Acupotomy for soft tissue disorder is a miniature surgery, with higher acceptability and less pain. It is crucial to make sure whether acupotomy is a good option for the patients, and whether it is as effective as local steroid injection. Local hormonal injection is a clinically widely used treatment for chronic soft tissue injury, which has a high degree of credibility and is often used in combination with needle knives in clinical practice. But it has some side effects and abuse. Studies have shown that acupotomy can effectively reduce the symptoms of soft tissue disorder, but its efficacy has not been evaluated scientifically and systematically. The aim of this study is to evaluate the efficacy and safety of the acupotomy treatment in patients with soft tissue disorder Whether it is the same as or better than local injection, whether it is superior to local hormone injection in terms of safety, side effects and recurrence rate, we hope this review will provide more evidence. To confirm the role of the needle knife, as an alternative to local hormone injection. There are some limitations in this review. Different types of acupotomy and degree different parts of the body may run the risk of heterogeneity. In addition, the measurements and tools of outcomes of included studies may be different.

## Author contributions

HS is the guarantor of the article. The manuscript was drafted by YS and TC. TL and JZ developed the search strategy. TC and TL will independently screen the potential studies and extract data. JZ and JG will assess the risk of bias and finish data synthesis. HS will arbitrate any disagreement and ensure that no errors occur during the review. All review authors critically reviewed, revised, and approved the subsequent and final version of the protocol.

## Supplementary Material

Supplemental Digital Content
