# Supplementary material for: A comparison between acupotomy vs the local steroid injection for the management of soft tissue disorder: A systematic review protocol
Source: Medicine (Baltimore). 2019 Nov 11;98(45):e17926. doi: 10.1097/MD.0000000000017926 (PMC6855588; doi:10.1097/MD.0000000000017926)
Supplement: Supplemental Digital Content [file medi-98-e17926-s001.doc]

**Appendix 1. Search strategy used in PubMed database**

1."Muscle"[Mesh] OR "[Tendinopathy](https://www.ncbi.nlm.nih.gov/mesh/68052256)"[Mesh] OR "[Ligaments](https://www.ncbi.nlm.nih.gov/mesh/68008022)"[Mesh] OR "[Synovial Membrane](https://www.ncbi.nlm.nih.gov/mesh/68013583)"[Mesh] OR "[Arthritis](https://www.ncbi.nlm.nih.gov/mesh/68001168)"[Mesh] OR "fascia"[Mesh] OR "joint"[Mesh] OR "tendon"[Mesh]

2. “Soft tissue disorders"[Title/Abstract] OR “[Myofascial Pain Syndromes](https://www.ncbi.nlm.nih.gov/mesh/68009209)"[Title/Abstract] OR “musculoskeletal pain”[Title/Abstract] OR “Synovitis” [Title/Abstract] OR “Tenosynovitis” [Title/Abstract] OR ”Tendinitis” [Title/Abstract] OR ”Tendinosis” [Title/Abstract] OR “Fasciitis”

3.#1 OR #2

4."pain"[Title/Abstract]

5.#3 AND#4

6."acupotomy"[Title/Abstract] OR "small needle-knife"[Title/Abstract] OR "needle knife"[Title/Abstract]

7. "local steroid injection"[Title/Abstract] OR "block therapy"[Title/Abstract] OR “corticosteroid injection”[Title/Abstract]

8.#6 AND #7

9. "randomized controlled trial"[Title/Abstract] OR "controlled clinical"[Title/Abstract]

7.5# AND #8 AND #9
